# Mechanochromic Fluorescent Polymers with Aggregation-Induced Emission Features

**DOI:** 10.3390/s19224969

**Published:** 2019-11-14

**Authors:** Andrea Pucci

**Affiliations:** Dipartimento di Chimica e Chimica Industriale, Università di Pisa, Via Moruzzi 13, 56124 Pisa, Italy; andrea.pucci@unipi.it; Tel.: +39-05-0221-9270; Fax: +39-05-0222-0673

**Keywords:** aggregation induced emission, polymers, mechanochromism, mechanochromic fluorescence

## Abstract

Mechanochromic polymers are defined as materials that are able to detect a mechanical stress through an optical output. This feature has evoked a growing interest in the last decades, thanks to the progress of chromogenic molecules whose optical characteristics and chemical functionalities allow their effective insertion in many thermoplastic and thermoset matrices. Among the different types of fluorogenic probes able to detect mechanical solicitations, those with aggregation-induced emission (i.e., AIEgens) have attracted tremendous interest since their discovery in 2001. In the present review, the main principles behind the AIEgens working behavior are introduced along with the current state of knowledge concerning the design and preparation of the derived mechanochromic fluorescent polymers. Examples are provided concerning the most ingenious solution for the preparation of chromogenic materials, starting from different types of commodity plastics or synthetic polymers and combined with the latest AIE technology to provide the most sensitive response to mechanical stress.

## 1. Introduction

In the last few years, fluorogenic and emissive compounds have created a new, thriving field of research devoted to the study and development of optically-active materials that are sensitive to different external stimuli [1,2,3,4,5]. Fluorescence was used for the first time as an analytical tool for the determination of various species and chemicals. Pretty quickly, sensing based on fluorescence has become a reference method for the detection of analytes or external perturbations, since specially designed emitting materials allow high sensitivity, selectivity and a high signal-to-noise ratio [6,7,8,9,10,11,12,13]. Nevertheless, the aggregation of aromatic planar fluorophores usually suffers from emission quenching phenomena in concentrated solutions and in the solid state, known as aggregation-caused quenching (ACQ), which strongly limits the number of accessible fluorophores for practical and effective applications also in the sensing field [14]. For example, planar perylene derivatives are reported as typical ACQ fluorophores, and showed limited applications if not properly modified to prevent their stacking behavior.

Notably, a revolutionary class of fluorescence materials featuring emission triggered by aggregation has received great attention in sensor applications since their discovery by Ben Zhong Tang in 2001 [15]. The effect, called aggregation-induced emission (AIE), arises from the restriction of fluorophore intramolecular motions (RIM) that are typical of those molecules whose structure consists of two or more units that can dynamically rotate against each other. 

Moreover the presence of a twisted propeller-shaped conformation renders intermolecular π-π interactions difficult in the aggregate state. Noteworthy, by allowing light emission in the aggregate and solid state, AIE fluorophores (AIEgens) show a striking impact on energy, optoelectronics, life science and the environment [16,17,18,19,20,21,22,23,24,25,26]. Fluorescent sensors based on the AIE mechanism take advantages from the ordinary fluorescent sensors, thanks to the very brilliant emission in the solid state that allows for the development of an efficient ON-OFF, and more interestingly, an OFF-ON optical response towards several interferences [27,28]. Recent reviews on the application of the AIE technology in the sensing field well evidence the active and growing interest of the scientific community on this topic [29,30,31,32,33,34,35,36,37,38,39]. As far as external solicitations are concerned, those derived by mechanical stress results are of the most investigated so far [40,41,42,43,44,45,46,47,48,49,50,51,52,53,54,55]. Mechanical forces are present every day in nature, and consist in pressure, shearing force, friction and stress, all of them possibly accountable for damages in macroscopic materials that are generally detectable with invasive or destructive sensing applications. For example, nature suggests that the color response towards external stimuli can be gathered both from the physical phenomena, such as diffraction and the interference of the incident light [56], and/or from the presence of dyes, fluorophores or pigments. Everybody knows that chameleons, cephalopods and lizards display impressive abilities to modulate the color of their skin in response to external stresses of various nature and origin [57]. Notably, color changes arise from physiological processes that influence the pigment distribution within the skin and favors the reorientation of reflective protein plates. The mechanism behind pigment transformation and relocation in biological tissues as a consequence of external stimuli, including also mechanical solicitations, have been inspiring the scientific community in the development of artificial, plastic, colored devices [58,59,60]. 

**Figure 1:**
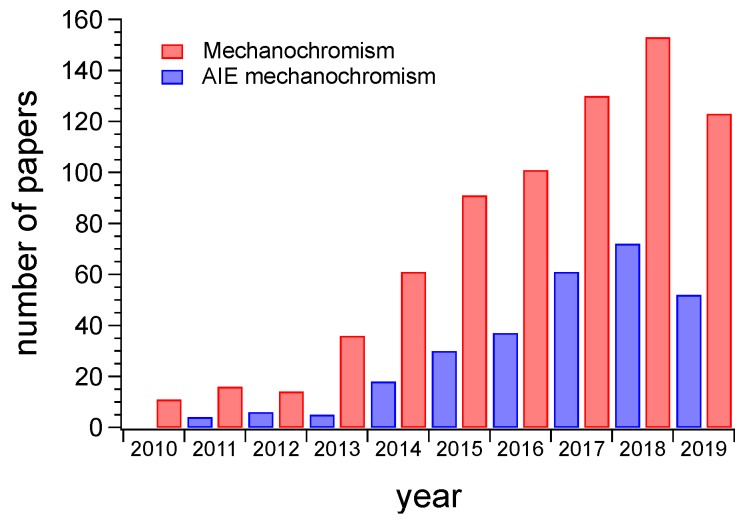
Number of papers published from 2010 onwards and related to mechanochromism (red histograms) and the same topic but promoted by the AIE technology (blue histograms). It is well evident the influence of the AIE research field on the development of mechanochromic fluorescent materials. (Data searched from Scifinder ® in July 2019).

The detection of mechanical stress through optical variations, referred to as mechanochromism, have therefore gained great importance also in the AIE technology field, since it enables the identification of the failure of in situ materials also at an early stage of damage, thus suggesting applications as anti-counterfeit systems and self-diagnostic materials (Figure 1) [41,61,62,63]. Being fluorescence, the optical output mechanochromism should be therefore intended as mechanochromic fluorescence.

In this respect, optical responses towards mechanical stimuli can be greatly amplified upon changes in the aggregation extent of AIEgens in the polymeric matrix due to variations of possible interphase interactions and supramolecular conformations, which likewise occurred for traditional chromogenic compounds [11,12,64,65,66]. Both dispersed and covalently-linked fluorophores can be proposed and designed as mechanically-responsive probes in polymers. 

Notably, in our earlier review paper on mechanochromic fluorescent polymers, we eventually summed up that the dispersion of chromogenic probes within polymers is certainly a preferred procedure for commodity polymers, even supposing that efforts are needed to prevent fluorophore segregation [10,11,12]. Conversely, the covalent bonding of chromophoric units with the macromolecular chains has the advantage of obtaining a material with homogeneously-distributed fluorescent probes whose molecular diffusion and eventual segregation is mostly prevented with time and use [12]. Nevertheless, the synthetic approach is however hindered by the need for properly designed reactive moieties on both macromolecular chains and fluorescent probes. 

Notwithstanding the enormous progress of the AIE research in the sensing field, a summary of the latest research devoted to the mechano-responsive characteristics of AIEgen probes incorporated in polymers is missing. Therefore, this review paper focuses on the description of AIEgens utilized as chromogenic probes in mechanochromic fluorescent polymer sensors. The main mechanism behind the chromogenic response of AIEgens as mechanochromic probes is discussed in terms of molecular design and incorporation strategy into polymers. Notably, examples of sensing devices obtained from the physical dispersion and the covalent approach of AIEgens in polymers are proposed and examined with regard to sensor response and performances. This review is conceived to provide the reader with illuminating information about these novel smart materials aimed to furnish useful tools to design and develop chromogenic AIEgens with even more sensitive response towards mechanical stimuli. 

## 2. AIE and Mechanochromism

**Figure 2:**
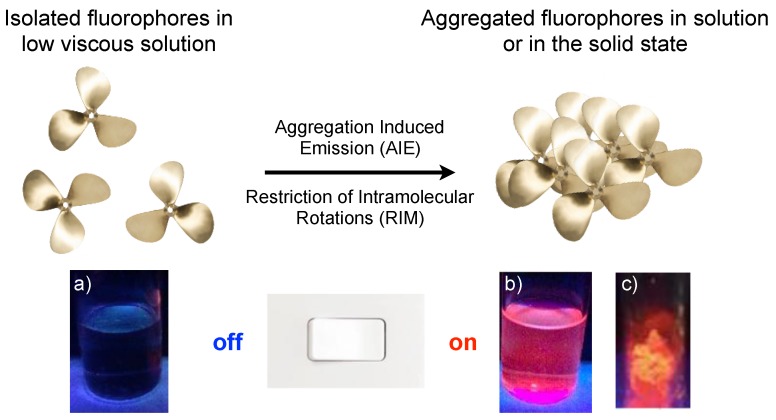
Working principle of the AIE mechanism: non-planar fluorophores start to emit light by aggregate formation, due to the restriction of the intramolecular rotation (RIR) of the multiple phenyl rotors against the stator in the aggregate state. Below are photos taken by exciting a red emissive AIE fluorophore respectively: (**a**) in a diluted solution of a good solvent; (**b**) in a solvent-non solvent mixture at the same molecule concentration; and (**c**) in the solid state. Reprinted with permission from [25]. Copyright (2018) John Wiley and Sons.

As mentioned earlier, the AIE effect arises from the restriction of intramolecular motions (RIM) of propeller-like fluorophores (Figure 2). The propeller shape is then reported to effectively disable the stacking interactions between chromophores, thus limiting the ACQ phenomenon, and in turn, endorsing bright luminescence in the aggregate and solid state. 

The intense luminescence coming from supramolecular aggregates triggered the research in the field of mechanochromic and piezochromic fluorescent materials, since the color of the emission could possibly change by external compression or mechanical grinding. Recently, many reports have been published on the effective mechanism of piezochromic fluorescence based on the molecular aggregation state of AIE luminogens [61,62,63,67,68,69,70,71,72,73,74,75,76,77,78,79,80,81,82,83,84,85,86]. 

Many AIEgens actually show crystallization-induced emission or crystallization-enhanced emission, and a mechanical solicitation applied to the chromophoric ordered structures possibly promotes the deaggregation of the fluorescent units. This process favors the reduction of the barrier energy associated with the AIEgen rotors, thus ending up with the typical ON-OFF behavior [61]. Even more interesting, if the mechanical stress is provided by compression or pressurization to amorphous conformations of AIEgens with loosely packing patterns, an enhanced emission intensity occurs due to crystallization phenomena triggered by the increased molecular interactions [87]. Therefore, the more attractive OFF-ON behavior is provided.

**Figure 3:**
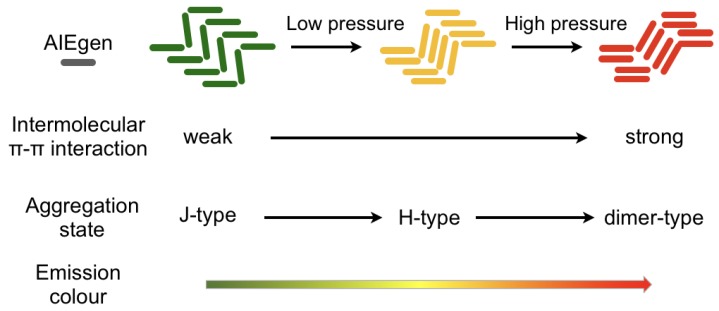
Schematic diagram of stacking mode and emission color with various molecular aggregation states in a generic AIE luminogen. Adapted with permission from [89]. Copyright (2013) John Wiley and Sons.

An intriguing solution aimed at providing a more sensitive fluorogenic response is provided in literature, profiting from the accessible modification of the AIE core with push–pull functional groups. The several stacking modes in such AIE functionalized crystals result in the formation of a J- or H-aggregate that are characterized by different luminescence features and affected by the diverse supramolecular state. Notably, the closer the two or more neighboring molecules are, the stronger the intermolecular π-π interactions among them, and also the exciton coupling and orbital overlap between chromophores [88,89]. It is actually reported that interactions between the excited state of an aromatic molecule and the ground state of the same molecule give rise to the excimer (excited dimer) formation, whose fluorescence is characterized by an unstructured emission and at a lower energy than the corresponding monomer emission [90]. Therefore, the efficient vibronic coupling of the AIEgen interchromophore breathing modes promoted by the occurrence of the strong intermolecular π-π interactions, is responsible for the significantly red-shifted and unstructured ‘excimer-like’ emission. This feature is effectively reflected on the optical properties of solid aggregates, which definitely changes accordingly (Figure 3). This chromogenic behavior enabled the publication of several papers devoted to the mechanochromic luminescence triggered by pressure and rubbing solicitations [61]. 

Very recently, further discussion has been addressed for the first time to red emissive carbon dots (R-CDs) that showed pressure-triggered aggregation-induced emission enhancement [80]. Notably, aromatic rings in the R-CDs are reported to undergo dynamic intraparticle oscillations, which cause the relaxation from the excited state in a non-radiative decay pathway. Noteworthy, compression greatly impeded these vibrations due to the physical constraint, thus favoring luminescence emission. 

Following this scenario, AIEgens are enabled to act as fluorescent probes to visualize the structural and morphological variations occurring in materials due to any possible contamination event that possibly changes the extent of their aggregation. On this account, the next session is devoted to the exploitation of the mechanochromic fluorescence of AIEgens when incorporated into polymer matrices. 

## 3. AIE and Mechanochromic Fluorescent Polymers

In contrast to small AIE luminogens, macromolecules with AIE characteristics have been less investigated so far. Notably, AIE polymers have many advantages over low mass AIEgens, such as processability, easy functionalization, good thermal stability and possess structural complexity and phase ordering that can be easily modulated by external solicitations [53,91,92]. On this account, AIEgens can be also utilized as piezochromic fluorescent additives in commodity polymers. The AIE behavior possibly enables the formulation of a more sensitive mechanochromic response with respect to the traditional planar ACQ fluorophores [93]. 

**Figure 4:**
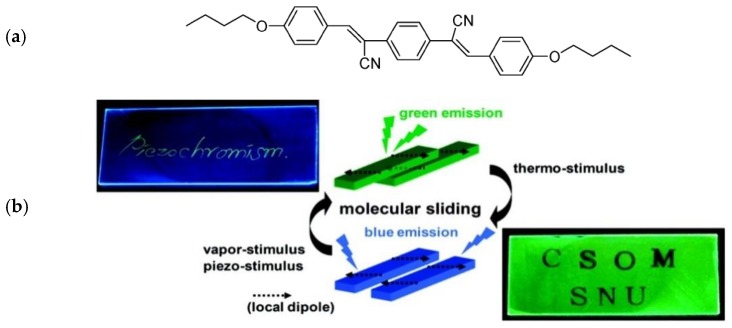
(**a**) Chemical structure of the cyano-substituted distyrylbenzene derivative; (**b**) Illustration of two different modes of slip-stacking in dye molecular sheets, dictated by different ways of the antiparallel/head-to-tail coupling of local dipoles and photos of the luminescence writing/erasing cycle of the dye/PMMA blend film. Reprinted with permission from [94]. Copyright (2010) American Chemical Society.

The first example reported in the literature concerning the application of polymers with AIE characteristics in the field of materials with a mechanochromic fluorescent response was reported by S.Y. Park et al [94]. Notably, a cyano-substituted distyrylbenzene derivative (Figure 4a) was synthesized to show the characteristic AIE character, i.e. a highly enhanced emission in the solid state than in the solution. Noteworthy, the authors found that the AIEgen interchanges in the solid state from a metastable green-emitting G-phase to a thermodynamically stable blue-emitting B-phase as a result of external stimuli, such as heat, pressure and the vapors of volatile organic compounds. Both solid-state analogs show very high fluorescence quantum yields (around 60%) owing to the AIE characteristics. The origin for the chromogenic emission is attributed to the different directional shear-sliding capability of molecular sheets that are promoted by different modes of local dipole coupling, i.e. antiparallel or head-to-tail, which cause a substantial alternation of π-π overlap. The chromic response in the solid state was elegantly utilized by the authors to prepare fluorescent polymeric films with a multichromic response. Notably, cyano-substituted distyrylbenzene/PMMA blend films with a thickness of 50 nm were obtained by vacuum deposition and displayed fast-responding and reversible multistimuli luminescence switching (Figure 4b). Indeed, upon heating the film at 125 °C for 10 s, the green emission at 536 nm shifted to blue at 458 nm in correspondence of the G- to B-phase transition of the AIEgen characterized by the head-to-tail coupling. The metastable green-emitting G-phase was then restored with the application of a mechanical solicitation, such as a shear force that promotes the antiparallel coupling of the local dipoles of the dye. The final exposure of the whole films to vapors of volatile organic compounds (i.e., CH_2_Cl_2_ for 30 s), definitely erases the entire emission color to green (Figure 4b).

Tetraphenylethylene (TPE), i.e. one of the classic building moieties of most of the investigated AIEgens [95], was then proposed as a lead character for the preparation of mechanochromic fluorescent polymer films following both the physically mixing and the covalent linkage approach. For example, TPE was firstly utilized in polymer films based on styrene-containing elastomers as reversible mechanochromic fluorescent devices. 

**Figure 5:**
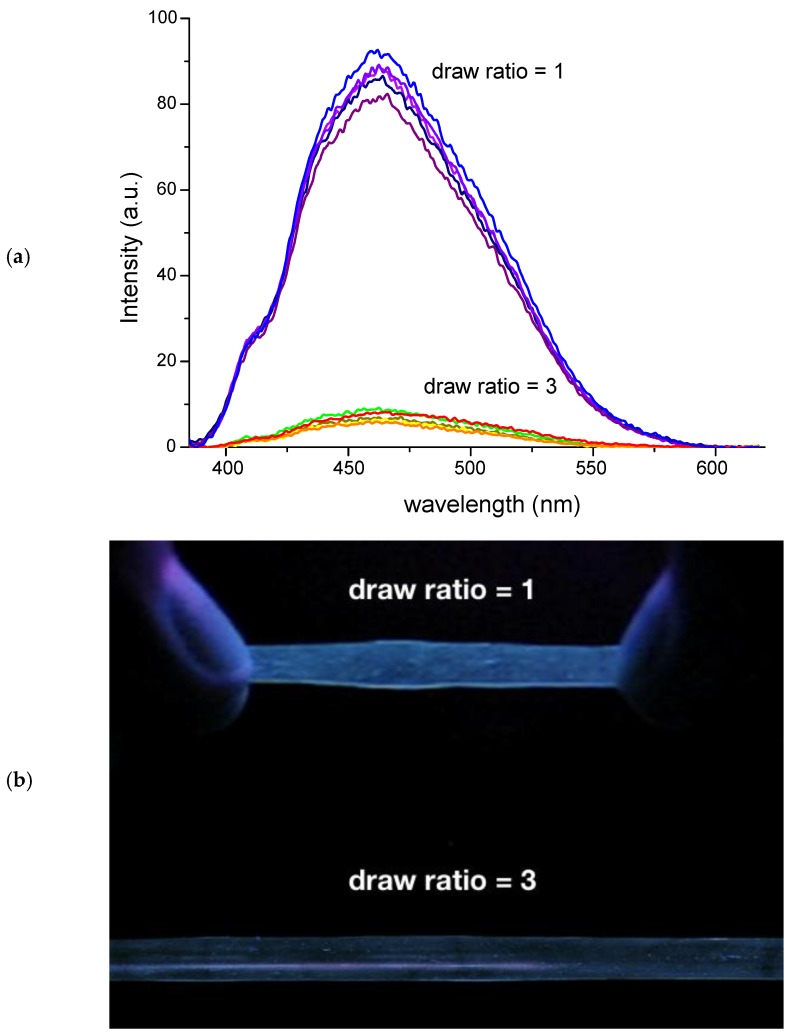
(**a**) Fluorescence spectra (λ_exc_ = 360 nm) of a 0.01 wt % of TPE/SBS film before (draw ratio = 1) and after (draw ratio = 3) uniaxial deformation at room temperature and (**b**) pictures of the same film under the irradiation at 366 nm. Draw ratio = 1 means the pristine, undeformed film.

This work was initially designed to determine the phase dispersion behavior of TPE in phase-separated domains of the poly(b-styrene-b-butadiene-b-styrene) (SBS) thermoplastic elastomer [96]. It was actually found that the TPE emission at room temperature resulted as being enhanced in SBS films. The block morphology of SBS actually enabled the presence of glassy, styrenic island-like phases characterized by more emitting features compared to the corresponding amorphous but no glassy character of the poly(styrene-co-butadiene) random (SBR) copolymer. SBS films containing the 0.01 wt % of TPE obtained by solution casting from chloroform mixtures were also found extremely responsive towards uniaxial deformation. As soon as the films experience mechanical drawing at a draw ratio higher than 1, the typical emission of TPE aggregated at about 460 nm collapses (Figure 5a), flanked by a clearly visible ON-OFF behavior, as detected by the irradiation with a long-range UV lamp at 366 nm (Figure 5b). 

This phenomenon was attributed to a combination of effects: the first resides on the change of thickness of the film upon drawing, which in turn decreased the concentration of the dye in the longitudinal axis direction of the excited area; the second involves the deaggregation of TPE units that promotes the reduction of the barrier energy associated to the phenyl rings rotation, thus leading to the drop in the emission intensity. 

**Figure 6:**
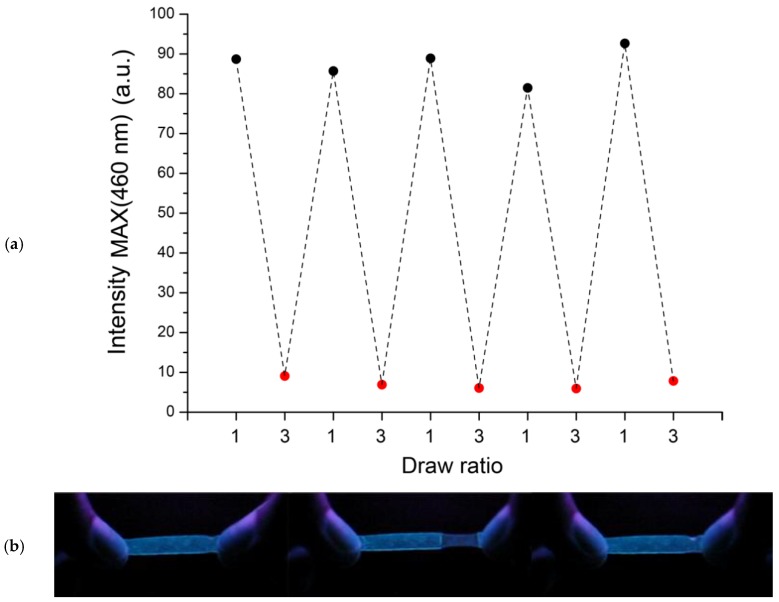
(**a**) Fluorescence intensity at 460 nm of a 0.01 wt % of TPE/SBS film before (draw ratio = 1) and after (draw ratio = 3) uniaxial deformation at room temperature (λ_exc_ = 360 nm) for different drawing cycles, and (**b**) pictures of the same film before (left), during (middle) and after deformation (relaxation, right) under the illumination with a long range UV lamp at 366 nm. Draw ratio = 1 means the pristine undeformed film.

More than that, profiting of the elastomeric features of the SBS polymer matrix, the responsive character was also found reversible. The film subjected to several drawing cycles perfectly restored the original emission once the mechanical stress was removed (Figure 6a).

This phenomenon is visible enough by the naked eye under the excitation of a low cost UV long range lamp at 366 nm (Figure 6b), thus suggesting the application of SBS films containing small amounts of the classic TPE AIEgen as optical sensors for the determination of mechanical stress or external perturbations. Notwithstanding the detectable chromogenic response, the optical output is characterized by an overall poor contrast. This feature is in agreement with the fact that chromophoric aggregates result scarcely unaffected by the orientation of the amorphous phase during uniaxial deformation. Conversely, such aggregates result in being sensitive during the plastic deformation regime of semi-crystalline, thermoplastic polymers [97]. The macromolecular slippage of the crystalline region triggers an aggregates break up, thus leading to clear emission color changes. 

Therefore, in order to overcome this issue and to provide well-defined optical changes also in amorphous polymer matrices, Kokado et al. proposed a new AIE elastomer based on poly (dimethylsiloxane) (PDMS) and TPE, that was covalently incorporated in the polymer matrix [98]. The different strategy aimed at the incorporation of the AIEgen via a covalent approach and allowed a strict control of the AIEgen content. Moreover, it improved the phase stability of the luminescent probe towards different solicitation cycles. A tetravinyl AIE luminogen based on TPE was actually synthesized and then reacted at different content (up to 7.8 wt %) with H-terminated PDMS via hydrosilylation to gather AIE elastomers. The obtained films showed the typical elastomeric behavior as revealed by tensile test, while the mechanical properties varied in agreement with the chain length of PDMS and crosslinking degree. 

**Figure 7:**
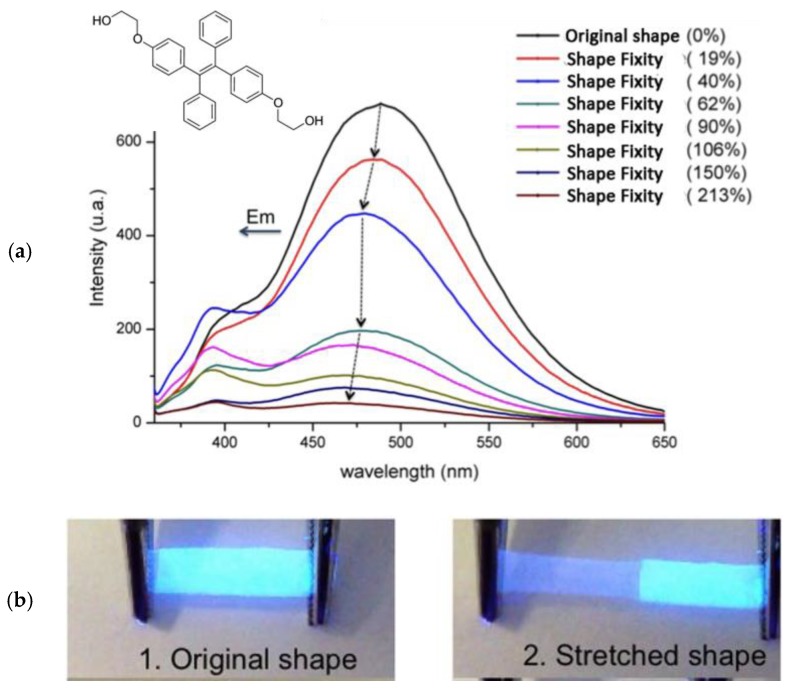
(**a**) Fluorescence spectra (λ_exc_ = 343 nm) of polyurethane films with 0.1 wt % of covalently-linked TPE as a function of the drawing ratio, and the chemical structure of the synthesized AIE probe in the inset; (**b**) the same film under the 365 nm UV lamp at a different stretching state. Adapted with permission from [99]. Copyright (2013) John Wiley and Sons.

Notwithstanding their elastomeric character, the authors reported the chromogenic behavior only in terms of the reversible response towards the exposure of volatile organic compounds and temperature variations. The same approach was however adopted by Tang et al. [99], who covalently incorporated TPE derivatives into polyurethane elastomers for the development of shape memory polymers and mechanochromic fluorescent materials with better contrast than that proposed earlier by using SBS. The luminescent polymers were realized by reacting poly(ε-caprolactone)diol (PCL, M_n_ = 4000) with 1,6-hexamethylene diisocyanate (HDI) and 1,4-butanediol (BDO) as diisocyanate and a chain extender to form a hard segment (about 25%). As a luminescent probe, a TPE-diol (Figure 7a, inset) was synthesized and directly connected to the polymer backbone at different content, i.e. from 0.02 to 0.1 wt %. Polymer films with a thickness of 0.1 mm were then obtained by casting from DMF polymer solutions and solvent evaporation at 70 °C for 12 h. As analogously reported in TPE/SBS blend films, the emission green band around 479 nm experienced a strong decreasing in intensity with the progression of the uniaxial film deformation up to 213% (Figure 7a). The fluorescence drop was also accompanied by a slight original blue shift from 479 to 468 nm after stretching and the decrease of emission lifetime from 1.32 ns to 0.89 ns at this stretched state. All these phenomena were addressed to morphology changes of TPE aggregates. De-aggregation of the AIEgen supramolecular arrangements was reported to occur during the stretching process. Notably, the emission intensity of polymeric film dropped dramatically before 100% shape fixity, and the phenomenon was clearly visible also by the naked eye under the excitation with a long-range UV light (Figure 7b). 

The examples that have been proposed so far are related to the mechanochromic fluorescent response with the typical ON-OFF behavior. This feature is not considered so straightforward by the scientific community, since the optical response appears sometimes scarcely detectable by the naked eye.

A modern solution for the development of mechanochromic fluorescent devices based on the OFF-ON mechanism was elegantly proposed by Moore et al., who took advantages of the core-shell microcapsules technology [100]. This strategy relies on one-component design, whose mechanochromic fluorescent response is not triggered by intermolecular interactions being based on the encapsulation chemistry. Therefore, the approach results in being highly accessible for a variety of devices due to the facile incorporation of microcapsules into existing materials and an already accessible reservoir of efficient AIEgens. Notably, the core-shell microcapsules were prepared according to the authors’ previously reported procedure and based on a single-batch, in situ emulsification condensation polymerization method [101], as analogously adopted by Weder et al. by using conventional aggregachromic fluorophores, such as cyano-substituted oligo (p-phenylene vinylene) [102]. Specifically, a nonfluorescent 1 wt % solution of TPE solution in hexyl acetate was encapsulated into double-walled polyurethane/poly(urea-formaldehyde) microcapsules with a diameter of 112 ± 10 μm and excellent thermal stability up to 220 °C. Transparent epoxy coatings incorporating 10 wt % of TPE microcapsules were prepared, and no emission (OFF state) emerged from the pristine composite when exposed to a long-range UV lamp. As soon as a mechanical damage occurs by scratching the surface of the doped epoxy resin with a razor blade, rupture of the microcapsules occurs, thus provoking the leakage of the encapsulated solution in the corresponding area of failure. The hexyl acetate evaporation causes the aggregation of TPE molecules and the rapid emersion (ON state) in the damaged regions of its typical fluorescence at about 450 nm with maximum intensity reached after about 5 min in ambient conditions. 

**Figure 8:**
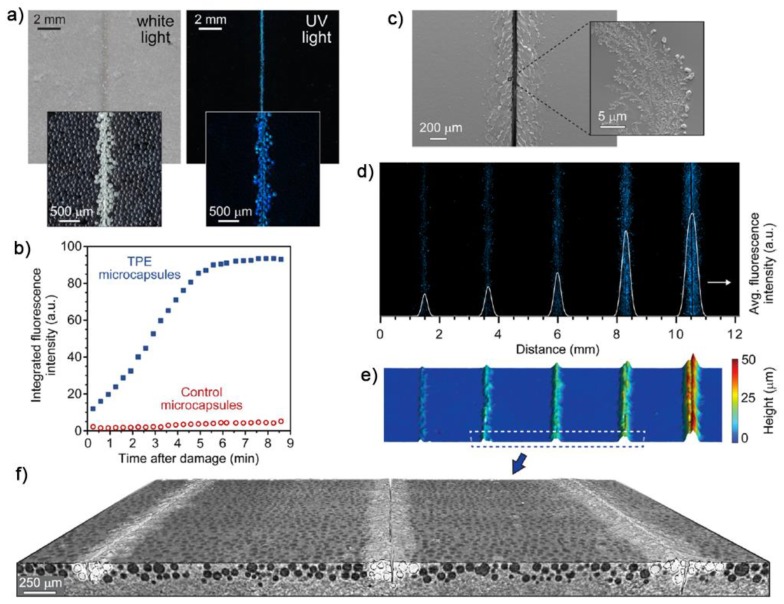
Evaluation of damage detection in encapsulated AIE epoxy coatings: (**a**) micrographs of an epoxy resin containing 10 wt % TPE containing microcapsules under illumination with white light and UV light after being scratched with a razor blade. Insets show stereomicrographs of the material under similar illumination; (**b**) time-dependent fluorescence microscopy illustrating the emersion of fluorescence after damage franked by a control experiment by using microcapsules with only hexyl acetate in the core; (**c**) SEM micrographs showing solid TPE deposits in the shear region close to the damage; (d–f) investigations of the epoxy resin doped with 10 wt % of TPE microcapsules with damages of varying size (i.e., 94, 140, 171, 222 and 376 μm, respectively): fluorescence micrograph and overlaid fluorescence intensity profile (**d**), surface topology from profilometry (**e**), and magnified view of a 3D micro-CT reconstruction with intact microcapsules rendered as black (**f**). Reprinted with permission from [100]. Copyright (2016) American Chemical Society.

The phenomenon was clearly visible under exposure to UV light and addressed by stereomicroscopy and control experiments to the fluorescence response of individually broken microcapsules (Figure 8). Since the area outside of the solicitation maintain its OFF state, excellent resolution and image contrast was obtained, and with a fluorescence intensity that accordingly depends upon the TPE concentration and incorporation of more microcapsules. This feature allowed also for a quantitative assessment of the extent of the mechanical solicitation. The accessibility of the proposed methodology to other classes of materials was also demonstrated by the authors on passing from thermoset materials such as epoxy resins to thermoplastic ones like polystyrene, acrylic polymers and PDMS. 

**Figure 9:**
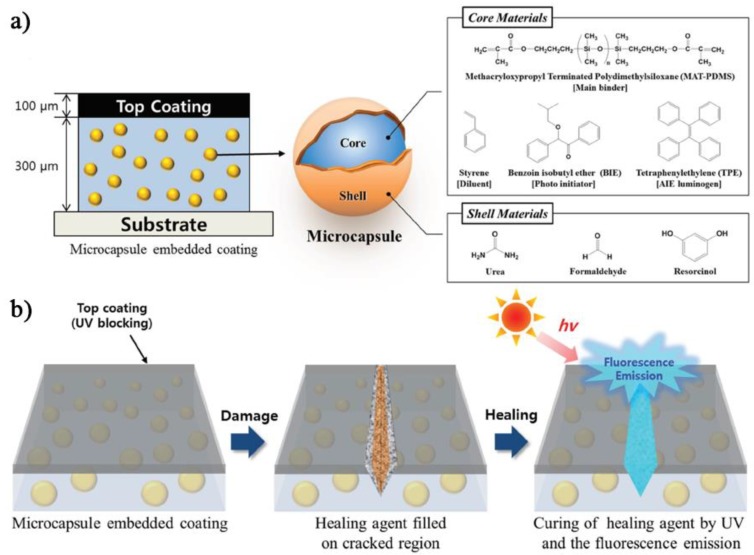
(**a**) Configuration of the coating containing embedded self-healing microcapsules and a schematic diagram of the microcapsule composition. (**b**) Schematic diagram of self-healing detection with fluorescence emission. Reprinted with permission from [103]. Copyright (2017) John Wiley and Sons.

Following this general idea, Young II Park et al. proposed to associate the mechanochromic fluorescence provided by the encapsulated TPE AIEgens with the extrinsic self-healing features in epoxy resins [103]. The core healing agent encapsulated in the urea-formaldehyde shell comprised in this case of a blend composed by methacryloxypropyl-terminated polydimethylsiloxane, styrene, benzoin isobutyl ether and TPE (Figure 9). Once the mechanical stress is applied to the epoxy matrix through a razor blade, the long-range UV lamp utilized for the detection of the damaged regions served also to activate the benzoin isobutyl ether (photoinitiator) and to promote the copolymerization between the styrene monomers and the siloxane crosslinker bearing the methacrylic reactive terminal units. The authors demonstrated that the storage modulus of the healing agent increased up to 10^5^ Pa after curing and that the blue emission provided by the released TPE increased during photocuring, and such enhancement was directly associated with an increase in the modulus of the epoxy matrix.

In a follow-up paper, the same authors proposed a system characterized by an extrinsic self-healing coating to identify cracked and healed regions by their yellow fluorescence and blue fluorescence, respectively [104]. Notably, the sensing system was based on a top UV light blocking layer, the epoxy resin containing the yellow light-emitting dye, and the microcapsules containing the healing agent, the photo-initiator and TPE. As soon as the damage occurs, the cracks are evidenced by the yellow fluorophore. Then, the blue AIE emission of the healed region emerged according to the irradiation time. 

**Figure 10:**
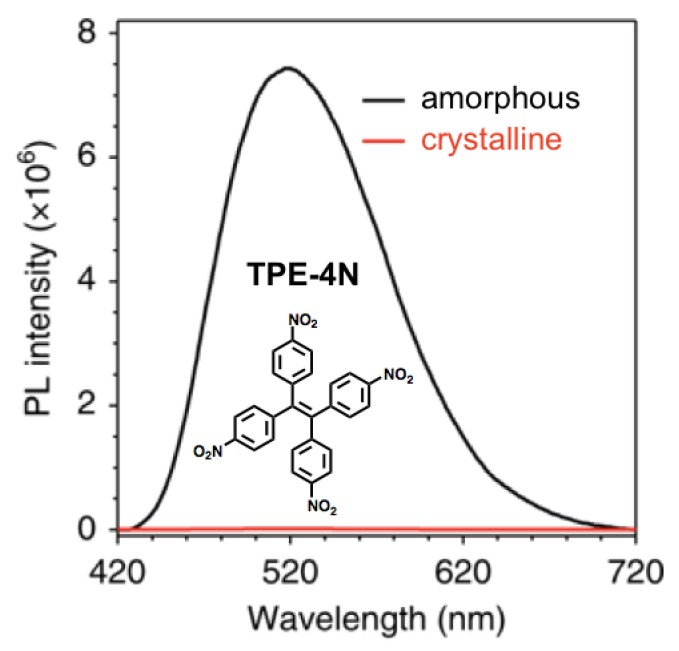
Emission spectra of 1,1,2,2-tetrakis(4-nitrophenyl)ethene (TPE-4N) in amorphous and crystalline states (λ_exc_ = 365 nm). Adapted with permission from [105]. Copyright (2018) Springer Nature.

Very recently, Tang et al. have proposed 1,1,2,2-tetrakis(4-nitrophenyl)ethene (TPE-4N, Figure 10) as a versatile AIEgen with an emission ultrasensitive to mechanical stimulus [105]. 

In this case, the discovered ON-OFF luminescence switching was addressed to the intersystem crossing (ISC) relaxation process from the excited singlet state to the highly sensitive triplet state, which is usually characterized by its nonemissive character. Experimental investigations reported that TPE-4N in the crystalline phase, the nitrophenyl groups effectively enable the nonradiative ISC channel to quench the emission. Computational analysis confirmed that the crystalline state favors the close proximity of the S_1_ state to the T_3_ state, thus promoting the occurrence of efficient ISC. Conversely, in the amorphous state, the TPE-4N luminogen recovered its luminescence with a bright green emission peaked at 520 nm, thus suggesting that the ISC relaxation process is hampered due to the AIE morphology change. The presence of the twisted molecular conformation in AIEgens was reported to favor ON-OFF luminescence switching in the solid state. 

Profiting from these chromogenic characteristics and the capability of TPE-4N to form homogeneous films over different surfaces, the same authors proposed its use as a mechanochromic fluorescent coating for the detection of stress–strain solicitations of metal specimens [106]. Regardless of the fact that the chromogenic AIEgen was not utilized in combination with a synthetic polymer, the coating technology is the same of those proprietary of common polymeric resins. Therefore, the authors’ contribution is anyway proposed in this review, being illustrative of a general working and effective procedure. In detail, a uniform 1 μm thick TPE-4N film was realized over a steel tensile specimen by dip-coating from a chloroform solution. 

**Figure 11:**
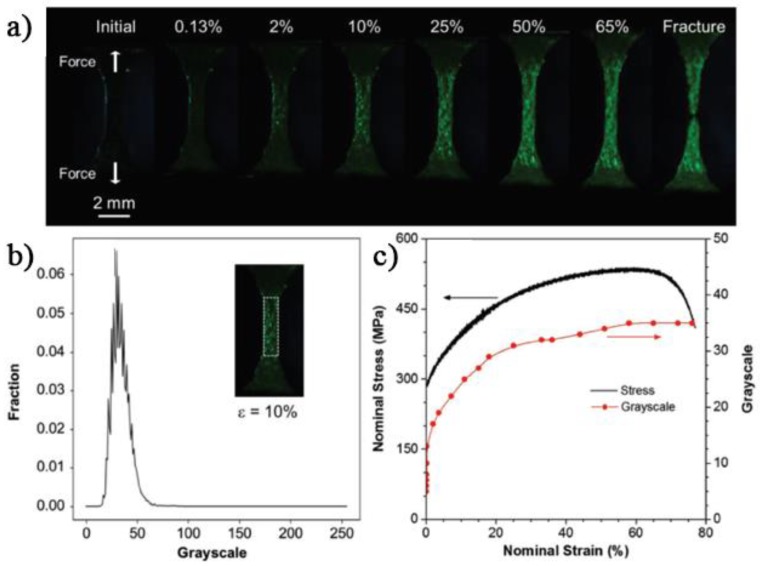
(**a**) Fluorescence images of TPE-4N-coated steel tensile specimen at different strains (ε%). Direction of stretching force: vertical; (**b**) grayscale distribution of the selected area at ε = 10%. Inset: Fluorescence image of TPE-4N-coated steel tensile specimen at ε = 10% and the selected area for grayscale analysis. (**c**) Plots of strain against stress and grayscale of the TPE-4N-coated steel tensile specimen. Reprinted with permission from [106]. Copyright (2018) John Wiley and Sons.

After air drying, the TPE-4N film showed the typical strong green fluorescence at 520 nm thanks to the amorphous character of the coated AIEgen. The emission was then totally quenched upon annealing the specimen at 150 °C for 20 min that triggered the crystallization process of the TPE-4N coating that in turn promptly activated the nonradiative ISC process. The annealed specimen was utilized for the mechanochromic tests by means of an in situ fatigue testing machine. Notably, the fluorescence variation was detected with a CCD camera under the irradiation with a coaxial UV light. When a force was applied, green fluorescence was observed again from the coated specimen and emerged progressively as a function of the external strain (Figure 11). 

Since TPE-4N is a turn-on type mechanochromic probe, its emission was activated and detected even at a very moderate strain of ε = 0.13% and with a progressive enhancement up to deformations of 20%. It was worth noting that the green emission intensity at ε = 65% resulted seven times greater than the pristine undeformed coated metal. The experimental investigations attributed the origin of the OFF-ON mechanochromic fluorescence to the crystalline–amorphous phase changes of the AIEgen coating layer upon uniaxial drawing.

## 4. Conclusions

Polymeric materials which respond to mechanical stress through variations of their optical response are called mechanochromic. Mechanochromic polymers have been extensively investigated since the pioneering work proposed by Weder in 2002 [107] and funded on the color changes associated with the formation of chromogenic aggregates based on cyano-substituted oligo(p-phenylene vinylene). This approach has been also demonstrated in wide range of dye-doped polymers by using different classes of aggregachromic fluorophores [12]. The fluorescent mechanochromism involves the progressive disruption of the fluorogenic aggregates as a function of the external stress that is transferred by the polymer matrix to the dispersed aggregates, thus yielding the clear changes of the overall color of the mechanochromic fluorescent device. Notwithstanding the amount of progress that has been made during the last decades in the field of chromogenic polymers, various new and intriguing challenges remain unresolved. For example, even if the optical response towards mechanical stress was clear and effective, research is still much needed to find a new class of fluorogenic dyes, since many of them suffer of the typical aggregation-caused quenching (ACQ) behavior. This controversy strongly limited the sensitivity of the prepared materials, thus hampering their final utilization in everyday life. This critical issue has been figured out by the emersion of the revolutionary class of fluorophore that starts to emit radiation when constrained in an aggregate supramolecular state or in high viscosity environment. Opposite to the ACQ behavior, the aggregation-induced emission (AIE) molecules, called also AIEgens, offer unique alternatives to scientists to provide materials with the desirable properties in terms of device sensitivity also to moderate deformations. 

Since the concept of AIE was born in 2001, thanks to the intuition of Tang [15], a plethora of fundamental studies and practical applications have been developed in many different fields, from AIEgens for electronic devices, stimuli-responsive applications and for the visualization of physical processes, as recently reviewed by Liu [39]. The rapid turn-on/turn-off nature of the AIEgens as a function of external stimuli is nowadays exploited for the development of modern mechanochromic fluorescent polymers. The easy functionalization of the AIE core appears also very useful for the introduction of the chromogenic probe in both thermoplastic and thermoset polymer matrices by a covalent pathway. Dispersion into polymers is certainly a sustainable procedure and largely applied to commodity plastics, but the covalent approach has the advantage of preventing AIEgen segregation and diffusion, and also of conferring to amorphous elastomeric matrices clear fluorescent variations towards mechanical stimuli. 

Both ON-OFF and OFF-ON chromogenic behaviors were taken into account, albeit this latter was considered promising for the development of highly sensitive mechanocromic fluorescent polymers. AIEgen dissolution into core-shell particles was actually proposed as a modern method for the development of useful mechanochromic OFF-ON fluorescent response. The extreme sensitivity of the AIEgen core to the minimum deformation extent combined with their encapsulation within ductile microcapsules appear so far the best solution for the preparation of auto-diagnostic polymer systems with a ratiometric response that gives access to quantitative assessment.

The inspiring examples reported in this review, together with the growing knowledge on the photophysics of fluorophores, will stimulate the design of even more effective plastic tools for the development of sensors, probes and information displays. Innovation would be in the chemical transformation of the typical ACQ fluorophores into AIE emitters by functionalizing the planar chromophoric nuclei with twisted propeller-shaped moieties. It is therefore expected that these concepts might steer innovative smart and mechanochromic fluorescent materials to be used in everyday life.
